# Real‐world implications of nonbiological factors with staging, clinical management, and prognostic prediction in pancreatic ductal adenocarcinoma

**DOI:** 10.1002/cam4.4910

**Published:** 2022-06-05

**Authors:** Chao Wang, Haoda Chen, Xiaxing Deng, Wei Xu, Baiyong Shen

**Affiliations:** ^1^ Department of General Surgery, Pancreatic Disease Center, Research Institute of Pancreatic Diseases, Ruijin Hospital Shanghai Jiaotong University School of Medicine Shanghai China

**Keywords:** AJCC‐TNM staging system, nonbiological factors, pancreatic ductal adenocarcinoma (PDAC), SEER, survival

## Abstract

**Background:**

The American Joint Committee on Cancer (AJCC) tumor‐node‐metastasis (TNM) staging system focuses on traditional biological factors (BFs). The present study incorporates nonbiological factors (NBFs) into the AJCC‐TNM staging system in terms of the advanced clinical management and prognostic‐prediction accuracy of pancreatic ductal adenocarcinoma (PDAC).

**Methods:**

Eight thousand three hundred and thirty eligible patients with PDAC were obtained from Surveillance, Epidemiology, and End Results database between January 1, 2011, and December 31, 2015. Multivariate Cox proportional hazards regression analysis and Kaplan–Meier curves were used to testify the feasibility of cancer‐specific survival (CSS) prediction based on TNM‐NBF stages.

**Results:**

The large population‐based study demonstrated that NBFs (insurance status, marital status, county‐level median household income, and unemployment) were significant prognostic indicators (*p* < 0.005), and multivariate Cox regression analysis demonstrated that the NBF1 stage carried a 29.4% increased risk of cancer‐specific mortality than NBF0 stage (*p* < 0.001). The concordance index of TNM‐NBF stage was 0.755 (95% confidence interval: 0.740–0.769).

**Conclusions:**

The novel NBF stage was independently associated with CSS of PDAC. In addition, combining TNM with the NBF stage could provide better clinical management and prognostic‐prediction accuracy.

## Graphical abstract

This study incorporates nonbiological factors (NBFs) into the AJCC‐TNM staging system in patients with PDAC, and the novel TNM‐NBF staging system could provide better clinical management and prognostic‐prediction accuracy

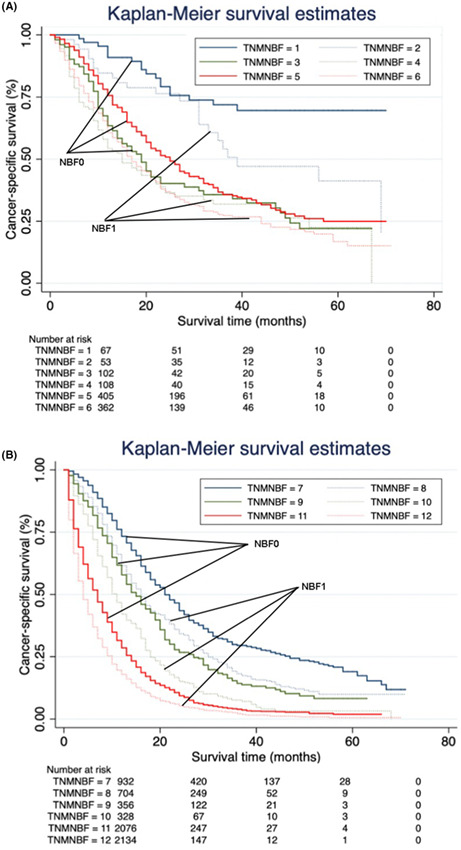

## 1 INTRODUCTION

Pancreatic ductal adenocarcinoma (PDAC) comprises 3% of newly diagnosed cancers and 8% of cancer‐related deaths and is estimated to contribute to over 48,220 deaths in 2021, a rate which has been annually rising.^1^ Although surgical resection remains as the predominant and optimal choice of therapy,^2^, ^3^ more than 70% of patients already have distant metastatic lesions or local vascular invasion, making them unqualified for direct radical surgery.^4^ Moreover, the prognosis remains poor even for patients who undergo pancreatectomy, owing to nodal involvement, and the high rates of recurrence.^5^ In this regard, the radical cure is seldom obtained for only 7%–10% of patients surviving 5 years after surgery.^6^, ^7^, ^8^ It is widely known that the prognosis of patients with PDAC is influenced by the tumor's innate behavior and natural history (biological factors [BFs]).^9^ Recent studies have shown that nonbiological factors (NBFs), such as health insurance status,^10^ marital status,^11^, ^12^ socioeconomic status,^13^ educational attainment,^14^, ^15^, ^16^ and unemployment status, were associated with the prognosis of patients with PDAC.

Since much attention has been given to the BFs of PDAC, including the extent of local invasion of the tumor (T stage), lymph node status (N stage), and distant metastases (M stage), the correlation between NBFs and the prognosis of PDAC has not yet been systematically demonstrated yet. It is not comprehensive to predict the prognosis of patients with PDAC only by the tumor‐node‐metastasis (TNM) staging system. In this respect, we use the data from the Surveillance, Epidemiology, and End Results (SEER) database to carry out a large population‐based study. We aimed to determine the role that NBFs play in the prognosis of PDAC and combine NBFs with traditional BFs to establish a novel TNM‐NBF staging system with regard to the more advanced clinical management and more accurate prognostic prediction of PDAC.

## 2 METHODS

### 2.1 Database and patient population

**FIGURE 1:**
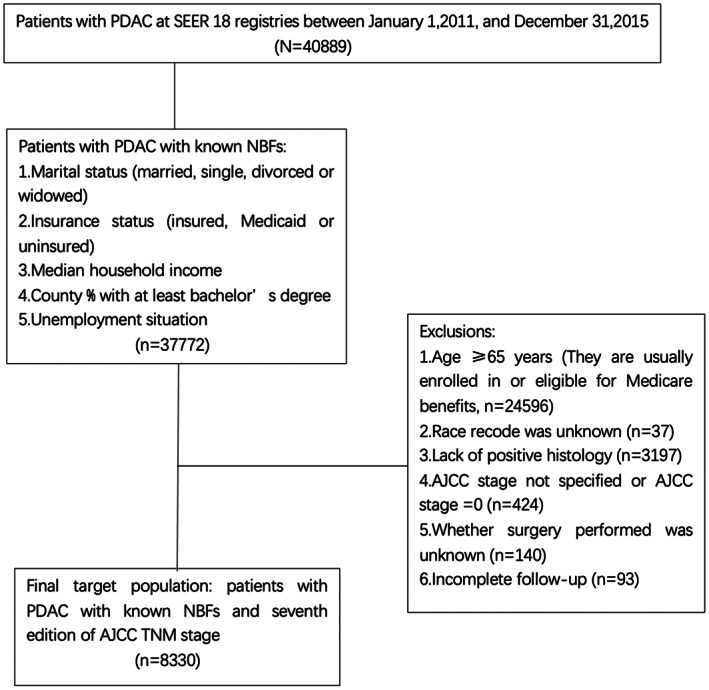
Flow diagram of patient population selected from SEER database. AJCC, American Joint Committee on Cancer; NBF, nonbiological factor; PDAC, pancreatic ductal adenocarcinoma; SEER, Surveillance, Epidemiology, and End Results; TNM, tumor‐node‐metastasis.

This study extracted information on patients diagnosed with PDAC from the SEER database. SEER is a database that is supported by the Surveillance Research Program in the National Cancer Institute's Division of Cancer Control and Population Sciences (DCCPS). We identified patients with pancreatic tumors (International Classification of Diseases for Oncology, 3rd edition [ICD‐O‐3], topographic code C25.0, C25.1, C25.2, C25.3, C25.7, C25.8, C25.9) diagnosed during 2011–2015. We then included patients with a histologic diagnosis of adenocarcinoma, defined by the ICD‐O‐3 morphologic codes 8140 and 8500. Initially, 40,889 patients were included in the present study. Those with known NBFs (including insurance status, and marital status, county percentage with a bachelor's degree, county‐level median household income, and unemployment status) were referred to this analysis. Insurance status was restricted to insured, Medicaid, and uninsured. Marital status was limited to married, divorced, single, and widowed. Patients with PDAC whose age was ≥65 years were excluded from the study because a majority of them were enrolled in or eligible for Medicare benefits. Finally, those of unknown race, lack of positive histology, unknown American Joint Committee on Cancer (AJCC) stage, unknown surgery status, and incomplete follow‐up were excluded, as shown in Figure 1.

### 2.2 Statistical analysis

All statistically meaningful and clinically relevant variables in univariate analysis were included in the multivariable Cox proportional hazards regression analysis, including the NBFs. All tests were two‐sided and performed at the 5% significance level.

**FIGURE 2:**
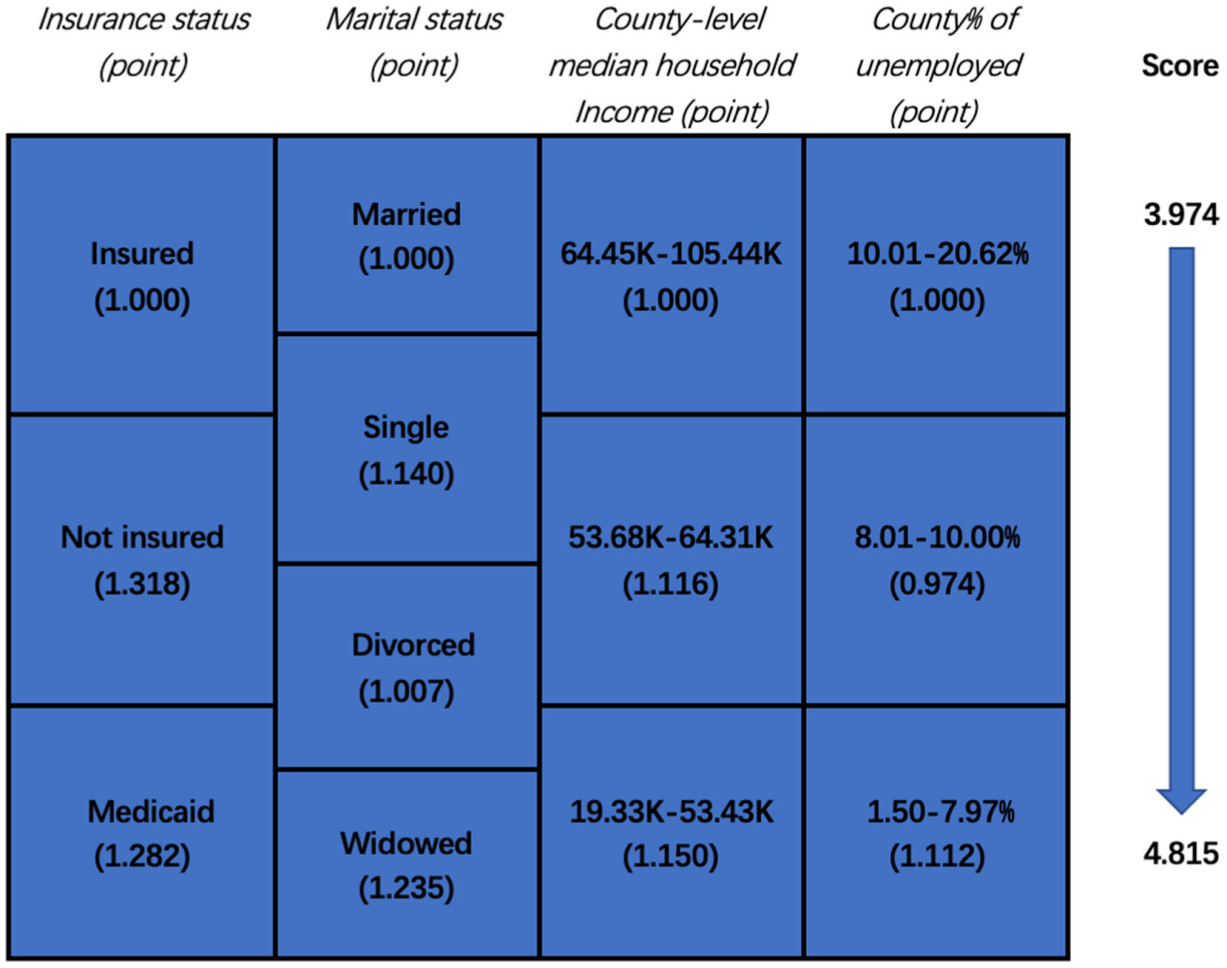
Patient prognostic score in pancreatic ductal adenocarcinoma: risk‐stratifications.

As shown in Figure 2, patients were stratified according to the different statuses of the four significant NBFs. The corresponding HRs, calculated by multivariate Cox regression analysis, were applied to assess the intrinsic roles NBFs plays in the prognosis of PDAC. The total prognostic score was then obtained by summing up the points of five NBFs of each patient. Then all populations were divided into two groups based on the median value of the scores. Patients with higher scores were assigned to stage NBF1, while the others were assigned to stage NBF0.

Baseline characteristics were compared using the Pearson chi‐squared test and Kruskal–Wallis rank‐sum test. The Kaplan–Meier survival curves were plotted from the time of diagnosis to the time of death or last follow‐up and were then compared using the log‐rank test. Then univariate and multivariate analyses were conducted to assess the veracity of the NBF stage and TNM‐NBF stage. The endpoint in the present study was cancer‐specific survival (CSS). All analyses were performed using Stata (Stata Corporation, version 15).

## 3 RESULTS

A total of 8330 patients with PDAC between January 1, 2011, and December 31, 2015, were obtained from the SEER Program database. The baseline characteristics of the patients with PDAC are presented in Table 1. The median follow‐up time was 39 (range, 0–71) months. In addition, 7017 (84.2%) patients had died of PDAC at the end of the follow‐up time.

**TABLE 1:** Baseline characteristics of patients with pancreatic ductal adenocarcinoma included in our study

Characteristics	No.(%)
Age (years)	
15–54	2573 (30.89%)
55–59	2464 (29.58%)
60–64	3293 (39.53%)
Sex	
Male	4637 (55.67%)
Female	3693 (44.33%)
Race	
White	6437 (77.27%)
Black	1237 (14.85%)
Others	656 (7.88%)
Year of diagnosis	
2011	1587 (19.05%)
2012	1631 (19.58%)
2013	1651 (19.82%)
2014	1672 (20.07%)
2015	1789 (21.48%)
American Joint Committee on Cancer‐tumor‐node‐metastasis stage	
IA	123 (1.48%)
IB	220 (2.64%)
IIA	789 (9.47%)
IIB	1668 (20.02%)
III	705 (8.46%)
IV	4825 (57.92%)
Insurance status	
Insured	6156 (73.90%)
Not insured	540 (6.48%)
Medicaid	1634 (19.62%)
Marital status	
Married	5107 (61.31%)
Single	1813 (21.76%)
Divorced	1112 (13.35%)
Widow	298 (3.58%)
County % with bachelor degree	
6.51%–24.80%	2783 (33.41%)
24.84%–34.66%	2794 (33.54%)
34.67%–55.77%	2753 (33.05%)
County‐level median household income	
19.33 K–53.43 K	2782 (33.40%)
53.68 K–64.31 K	2976 (35.73%)
64.45 K–105.44 K	2572 (30.87%)
County % were unemployed	
1.50%–7.97%	2784 (33.42%)
8.01%–10.00%	3498 (41.99%)
10.01%–20.62%	2048 (24.59%)
Surgery	
Surgery performed	2471 (29.66%)
Surgery not performed	5859 (70.34%)
Chemotherapy	
Yes	5989 (71.90%)
No/unknown	2341 (28.10%)

### 3.1 Four NBFs were closely related to the CSS of PDAC

Univariate analysis showed that age, sex, race, year of diagnosis, AJCC‐TNM stage, insurance status, marital status, county percentage with bachelor‘s degree, county‐level median household income, county percentage of unemployment, surgery status, and chemotherapy were associated with CSS (*p* < 0.2). These factors were then brought into multivariate Cox regression analysis. The data demonstrated that the following NBFs were independently associated with CSS: insurance status, marital status, county‐level median household income, and county percentage of unemployment (Table 2). Other related independent factors included age, sex, TNM stage, surgery status, and chemotherapy.

**TABLE 2:** Univariate survival analysis and multivariate Cox regression analysis of pancreatic ductal adenocarcinoma cause‐specific survival

Variable	Reference	Characteristics	Univariate analysis			Multivariate analysis		
			HR (95% CI)	*SE*	*p*	HR (95% CI)	*SE*	*p*
Age	15–54	55–59	1.071 (1.005–1.140)	0.034	0.033	1.079 (1.013–1.150)	0.035	0.018
		60–64	1.023 (0.964–1.085)	0.031	0.451	1.101 (1.036–1.169)	0.034	0.002
Sex	Male	Female	0.863 (0.821–0.906)	0.022	<0.001	0.898 (0.854–0.944)	0.023	<0.001
Race	White	Black	1.193 (1.114–1.279)	0.042	<0.001	1.035 (0.962–1.113)	0.038	0.359
		Other	0.949 (0.865–1.042)	0.045	0.275	1.010 (0.919–1.111)	0.049	0.831
Year of diagnosis	2011	2012	0.953 (0.883–1.023)	0.037	0.211	/	/	/
		2013	0.924 (0.855–0.997)	0.036	0.042	/	/	/
		2014	0.941 (0.871–1.017)	0.037	0.127	/	/	/
		2015	0.942 (0.868–1.021)	0.039	0.146	/	/	/
Tumor‐node‐metastasis stage	IA	IB	3.048 (2.153–4.317)	0.541	<0.001	2.623 (1.849–3.721)	0.468	<0.001
		IIA	2.755 (2.003–3.789)	0.448	<0.001	2.828 (2.052–3.896)	0.463	<0.001
		IIB	3.133 (2.294–4.280)	0.499	<0.001	4.069 (2.975–5.565)	0.650	<0.001
		III	4.923 (3.583–6.764)	0.798	<0.001	3.485 (2.520–4.821)	0.578	<0.001
		IV	9.751 (7.159–13.283)	1.538	<0.001	5.825 (4.241–8.000)	0.943	<0.001
Insurance status	Insured	Not insured	1.458 (1.315–1.615)	0.076	<0.001	1.318 (1.186–1.463)	0.071	<0.001
		Medicaid	1.497 (1.406–1.592)	0.047	<0.001	1.282 (1.200–1.369)	0.043	<0.001
Marital status	Married	Single	1.345 (1.265–1.430)	0.042	<0.001	1.140 (1.068–1.217)	0.038	<0.001
		Divorced	1.142 (1.062–1.232)	0.043	<0.001	1.007 (0.933–1.086)	0.039	0.863
		Widow	1.410 (1.240–1.605)	0.093	<0.001	1.235 (1.083–1.409)	0.083	0.002
County % with bachelor degree	34.67%–55.77%	24.84%–34.66%	1.075 (1.012–1.142)	0.033	0.019	1.014 (0.944–1.088)	0.038	0.708
		6.51%–24.80%	1.194 (1.124–1.268)	0.037	<0.001	1.050 (0.958–1.151)	0.049	0.301
County‐level median household income	64.45 K–105.44 K	53.68 K–64.31 K	1.107 (1.042–1.177)	0.034	0.001	1.116 (1.033–1.206)	0.044	0.005
		19.33 K–53.43 K	1.222 (1.149–1.299)	0.038	<0.001	1.150 (1.046–1.264)	0.055	0.004
County % were unemployed	10.01%–20.62%	8.01%–10.00%	0.878 (0.823–0.934)	0.028	<0.001	0.974 (0.904–1.049)	0.037	0.487
		1.50%–7.97%	0.890 (0.834–0.950)	0.030	<0.001	1.112 (1.030–1.201)	0.044	0.007
Surgery	Surgery performed	Surgery not performed	3.600 (3.389–3.823)	0.111	<0.001	2.645 (2.421–2.890)	0.120	<0.001
Chemotherapy	Yes	No/unknown	2.154 (2.034–2.282)	0.063	<0.001	2.279 (2.147–2.419)	0.069	<0.001

### 3.2 NBF stage was independently associated with the CSS of patients with PDAC

The sum of prognostic scores of NBFs ranged from 3.974 to 4.815 and a score of 3.974 represented optimal prognosis, while a score of 4.815 indicated the worst prognosis. Based on the median value of the prognostic scores, 8330 patients were finally divided into two groups: 4210 patients (50.54%) were designated to the NBF0 stage, and 4120 patients (49.46%) were designated to the NBF1 stage. Multivariate Cox regression analysis showed that NBF1 was independently related to the CSS of 8330 patients with PDAC, with a 29.4% increased risk of cancer‐specific mortality (HR: 1.294, 95% CI: 1.231–1.360, *p* < 0.001, Table 3). For a substantial proportion of patients who already have metastasis at their first diagnosis, we conducted a multivariate Cox regression analysis on metastatic and nonmetastatic PDAC. In comparison with the NBF0 stage, patients with nonmetastatic PDAC carried a 32.6% increased risk of cancer‐specific mortality in the NBF1 stage (HR: 1.326, 95% CI: 1.223–1.438, *p* < 0.001), which was notably higher than that in the general level (Tables 4 and 5). This result indicated that the efficacy of prognostic prediction based on the NBF stage was improved in patients with TNM stage I–III PDAC.

**TABLE 3:** Multivariable Cox regression analysis of independent prognostic factors in pancreatic ductal adenocarcinoma

Variable	Reference	Characteristic	Cancer‐specific survival		
			HR (95% CI)	*SE*	*p*
Age	15–54	55–59	1.073 (1.008–1.143)	0.035	0.028
		60–64	1.089 (1.026–1.156)	0.033	0.005
Sex	Male	Female	0.898 (0.855–0.944)	0.228	<0.001
tumor‐node‐metastasis	IA	IB	2.612 (1.842–3.705)	0.466	<0.001
		IIA	2.837 (2.059–3.909)	0.464	<0.001
		IIB	4.061 (2.969–5.554)	0.649	<0.001
		III	3.458 (2.501–4.783)	0.572	<0.001
		IV	5.795 (4.219–7.959)	0.938	<0.001
Surgery	Surgery performed	Surgery not performed	2.653 (2.429–2.897)	0.119	<0.001
Chemotherapy	Yes	No/unknown	2.319 (2.186–2.460)	0.070	<0.001
Nonbiological factor stage	Stage 0	Stage 1	1.294 (1.231–1.360)	0.033	<0.001

**TABLE 4:** Multivariate Cox regression analyses of independent prognostic factors in metastatic pancreatic ductal adenocarcinoma

Variable	Reference	Characteristic	Cancer‐specific survival		
			HR (95% CI)	*SE*	*p*
Age	15–54	55–59	1.062 (0.981–1.150)	0.043	0.140
		60–64	1.104 (1.024–1.191)	0.043	<0.001
Sex	Male	Female	0.893 (0.838–0.951)	0.029	<0.001
Surgery	Surgery performed	Surgery not performed	2.028 (1.707–2.408)	0.178	<0.001
Chemotherapy	Yes	No/unknown	2.415 (2.245–2.599)	0.090	<0.001
Nonbiological factor stage	Stage 0	Stage 1	1.252 (1.175–1.335)	0.041	<0.001

**TABLE 5:** Multivariate Cox regression analyses of independent prognostic factors in nonmetastatic pancreatic ductal adenocarcinoma

Variable	Reference	Characteristic	Cancer‐specific survival		
			HR (95% CI)	*SE*	*p*
Age	15–54	55–59	1.074 (0.968–1.191)	0.057	0.178
		60.‐64	1.040 (0.945–1.145)	0.051	0.424
Sex	Male	Female	0.920 (0.849–0.997)	0.038	<0.001
Tumor‐node‐metastasis	IA	IB	2.641 (1.861–3.748)	0.472	<0.001
		IIA	2.787 (2.019–3.847)	0.458	<0.001
		IIB	4.137 (3.019–5.668)	0.665	<0.001
		III	3.289 (2.371–4.564)	0.550	<0.001
Surgery	Surgery performed	Surgery not performed	3.118 (2.821–3.446)	0.159	<0.001
Chemotherapy	Yes	No/unknown	2.064 (1.864–2.285)	0.107	<0.001
Nonbiological factor stage	Stage 0	Stage 1	1.326 (1.223–1.438)	0.055	<0.001

### 3.3 Baseline analysis according to NBF stage

The Kruskal–Wallis rank test and trend test were applied to the baseline analysis of patients involved in our study. The results demonstrated that there were significant distinctions in almost all related variables between NBF0 and NBF1, except sex, with a *p* = 0.552 (Table 6). Then univariate survival analysis was carried out to determine the specific impact that the NBF stage has on CSS. We also analyzed the interaction between the NBF stage and other independent prognostic indicators. It is noteworthy that a significant difference could be found only in chemotherapy between the NBF0 stage and the NBF1 stage (*p* = 0.006, Table 7).

**TABLE 6:** Baseline analysis of 8330 patients with pancreatic ductal adenocarcinoma

Characteristics	NBF0 (*N* = 4210)	NBF1 (*n* = 4120)	*p*
Age			<0.001
15–54	1164 (27.65%)	1409 (34.20%)	
55–59	1256 (29.83%)	1208 (29.32%)	
60–64	1790 (42.52%)	1503 (36.48%)	
Sex (male)	2357 (55.99%)	2280 (55.34%)	0.552
Race			<0.001
White	3384 (80.38%)	3053 (74.10%)	
Black	428 (10.17%)	809 (19.64%)	
Other	398 (9.45%)	258 (6.26%)	
Tumor‐node‐metastasis			<0.001
IA	67 (1.59%)	56 (1.36%)	
IB	105 (2.49%)	115 (2.79%)	
IIA	410 (9.74%)	379 (9.20%)	
IIB	948 (22.52%)	720 (17.48%)	
III	362 (8.60%)	343 (8.32%)	
IV	2318 (55.06%)	2507 (60.85%)	
Surgery	1424 (33.82%)	1047 (25.41%)	<0.001
Chemotherapy	3324 (78.95%)	2665 (64.68%)	<0.001

Abbreviation: NBF, nonbiological factor.

**TABLE 7:** The interaction between the nonbiological factor stage and other independent prognostic indicators

Subgroups	HR (95% CI)	*p*_HR_	*p* for interaction
Overall	1.377 (1.311–1.447)	<0.001	
Age			
15–59	1.403 (1.317–1.495)	<0.001	0.508
60–64	1.340 (1.238–1.451)	<0.001	
Sex			
Male	1.408 (1.318–1.504)	<0.001	0.324
Female	1.351 (1.254–1.455)	<0.001	
Tumor‐node‐metastasis stage			
I–III	1.399 (1.291–1.515)	<0.001	0.921
IV	1.342 (1.260–1.428)	<0.001	
Surgery			
Yes	1.311 (1.185–1.451)	<0.001	0.431
No	1.324 (1.251–1.401)	<0.001	
Chemotherapy			
Yes	1.278 (1.207–1.353)	<0.001	0.006
No/unknown	1.316 (1.187–1.458)	<0.001	

### 3.4 Prognostic prediction of TNM‐NBF stage

ROC curves were plotted and the concordance index of the TNM‐NBF stage was 0.755 (95% CI: 0.740–0.769), which was higher than that of the TNM stage (HR: 0.744, 95% CI: 0.730–0.757). We used the Kaplan–Meier survivor function to analyze the prognostic‐prediction accuracy of the TNM‐NBF stage in the overall cohort (*n* = 8330). Further log‐rank *χ* ^2^ test was carried out to determine the significance of differences between the NBF0 stage and the NBF1 stage in each TNM stage. As shown in Table 8, most NBF0 stage patients carried a significantly increased CSS compared with NBF1 stage patients (*p* < 0.05) except IB stage patients, with a *p* = 0.415.

**TABLE 8:** Prognosis of TNM‐NBF stage in pancreatic ductal adenocarcinoma

TNM stage	NBF stage	TNM‐NBF stage	No. of patients	Univariate analysis		Multivariate analysis		
				Log‐rank	*p*	HR (95% CI)	*SE*	*p*
IA	NBF0	1	67	Reference		Reference		
	NBF1	2	56	5.05	0.025	1.860 (1.004–3.448)	0.586	0.049
IB	NBF0	3	105	Reference		3.460 (2.055–5.827)	0.920	<0.001
	NBF1	4	115	0.67	0.415	3.766 (2.245–6.316)	0.994	<0.001
IIA	NBF0	5	410	Reference		3.394 (2.099–5.489)	0.832	<0.001
	NBF1	6	379	15.04	<0.001	4.465 (2.763–7.214)	1.093	<0.001
IIB	NBF0	7	948	Reference		4.958 (3.098–7.933)	1.189	<0.001
	NBF1	8	720	33.13	<0.001	6.252 (3.903–10.014)	1.503	<0.001
III	NBF0	9	362	Reference		4.167 (2.575–6.744)	1.024	<0.001
	NBF1	10	343	22.92	<0.001	5.412 (3.341–8.765)	1.332	<0.001
IV	NBF0	11	2318	Reference		6.971 (4.351–11.168)	1.676	<0.001
	NBF1	12	2507	96.5	<0.001	9.084 (5.670–14.552)	2.184	<0.001

Abbreviations: NBF, nonbiological factor; TNM, tumor‐node‐metastasis.

**FIGURE 3:**
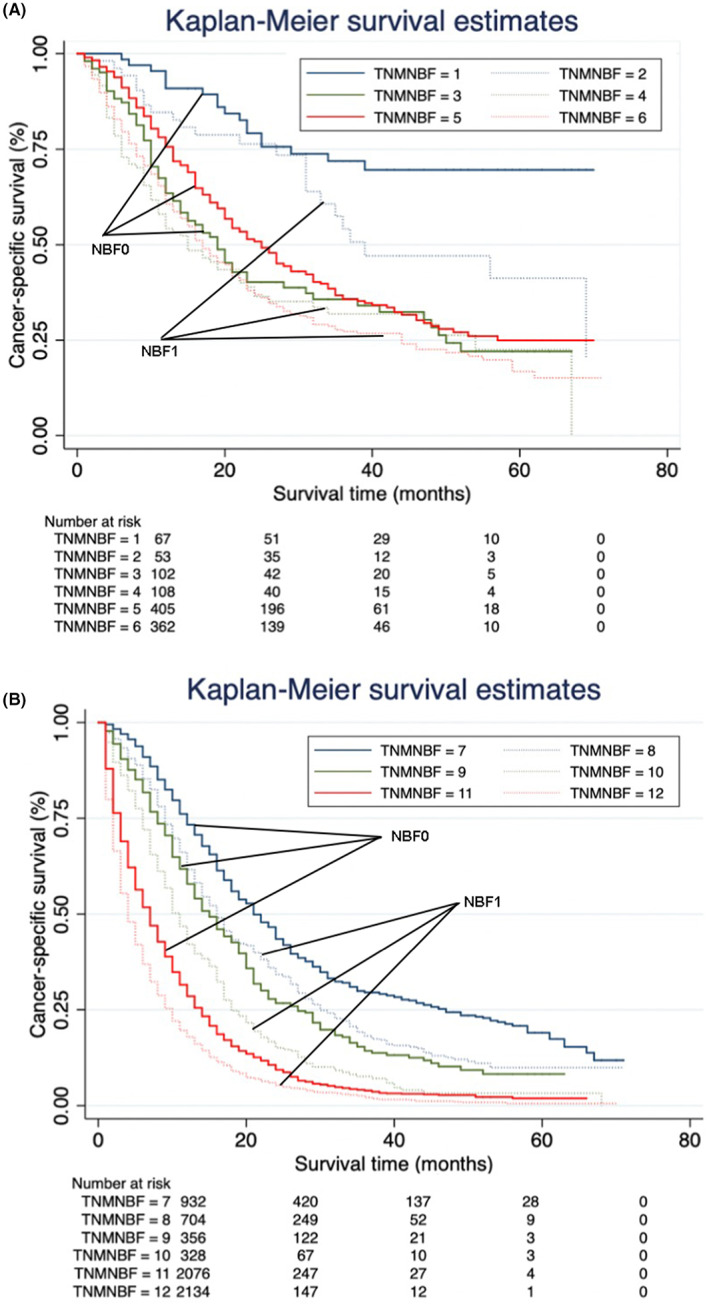
Kaplan–Meier survival curves of the TNM‐NBF staging system. (A) Cancer‐specific survival (CSS) of IA‐NBF0 stage, IA‐NBF1 stage, IB‐NBF0 stage, IB‐NBF1 stage, IIA‐NBF0 stage, IIA‐NBF1 stage. (B) CSS of IIB‐NBF0 stage, IIB‐NBF1 stage, III‐NBF0 stage, III‐NBF1 stage, IVNBF0 stage, and IV‐NBF1 stage. NBF, nonbiological factor; TNM, tumor‐node‐metastasis.

We then applied multivariate Cox regression analysis to assess the HRs of each TNM stage and TNM‐NBF stage. As was expected, all the TNM‐NBF0 stage patients carried lower HRs than the respective TNM‐NBF1 stage patients (Figure 3). It should be noted that the HRs of several NBF1 stage patients with lower TNM stages even exceeded those of NBF0 patients who displayed higher TNM stages. For instance, the cancer‐specific mortality was significantly higher in stage IB‐NBF1 patients (HR: 3.766, 95% CI: 2.245–6.316) than in stage IIA‐NBF0 patients (HR: 3.394, 95% CI: 2.099–5.489). The same phenomenon was observed in stage IIB‐NBF1 and stage III‐NBF0, with an HR decrease from 6.252 to 4.167 (*p* < 0.001). Moreover, when considering the same NBF stage, the HRs of the stage IIB‐NBF1 patients (HR: 6.252, 95% CI: 3.903–10.014) were higher than those of stage III‐NBF1 patients (HR: 5.142, 95% CI: 3.341–8.765).

## 4 DISCUSSION

Much attention is given to the biology of PDAC at the cellular and molecular levels and great progress has been made in those fields nowadays.^17^, ^18^ On the other hand, researchers are increasingly learning that NBFs, including insurance status, marital status, socioeconomic status, educational attainment, and unemployment, serve as an unneglected indicator of the prognosis of PDAC.^10^, ^11^, ^12^, ^13^, ^14^, ^15^, ^16^ Thorough evaluations of the clinical significance of NBFs have been made in the colon and rectal cancers.^19^, ^20^ However, the systematic study of all these NBFs in PDAC remains a field lack of exploration, not to mention the combination of the AJCC‐TNM system with NBFs.

There is growing recognition that insurance status is independently associated with CSS in several cancers.^21^, ^22^, ^23^, ^24^, ^25^ Compared with insured patients, patients without private insurance (those who are Medicaid or not insured) were significantly more likely to experience unfavorable survival. Similar conclusions were observed in patients with PDAC.^10^ We consider that patients without insurance are likely to receive delayed diagnosis and treatment, and those enrolled in the Medicaid program usually suffer from psychiatric or physical comorbidities, which account for the poor prognosis of PDAC.

Meanwhile, several studies have demonstrated that marital status is inextricably linked with CSS in the majority of cancers, including PDAC.^11^, ^26^, ^27^, ^28^, ^29^ As is shown in these studies, married people present with increased short‐term survival and decreased cause‐specific mortality compared with others. It is believed that married status seems to be beneficial to immune function and psychosocial health.^30^ Moreover, previous physiological researches have indicated that married status is closely associated with improvements in the cardiovascular and endocrine systems, thus leading to better cancer prognosis.^31^

Socioeconomic status, in addition, has also been proven to have a considerable impact on the overall survival of patients with several cancers.^32^, ^33^, ^34^, ^35^, ^36^ Unemployment and low household income act as strong predictors of unfavorable prognosis.^13^ It was also proven that patients with PDAC from more affluent areas were more likely to be diagnosed at an earlier stage and to receive timely surgery or accurate chemotherapy.^37^ Furthermore, a low education level has been identified to be a negative contributor to the overall survival of patients with PDAC.^14^, ^15^ Researchers have confirmed that low education levels are likely to result in psychological distress^38^, ^39^ and fear of tumor recurrence.^40^

The AJCC‐TNM staging system has limitations in that it mainly concentrates on the extent of local invasion of the tumor, lymph node status, and distant metastases, despite its widespread application globally. However, other factors including NBFs are not taken into consideration by the TNM staging system. It is inconclusive to determine the prognosis of patients with PDAC only on the TNM staging system.

Herein, we defined a new NBF stage (insurance status, marital status, county‐level median household income, and unemployment) to determine its prognostic‐prediction accuracy for PDAC. The present study indicated that the NBF stage was independently related to the CSS of patients with PDAC. The NBF1 stage patients carried a 29.4% higher risk of cancer‐specific mortality than NBF0 stage patients. Further studies indicated that a 25.2% and a 32.6% increased risk was observed in metastatic and nonmetastatic NBF1 stage patients respectively.

Moreover, we combined the TNM stage with the NBF stage and multivariate Cox regression analysis showed that the TNM‐NBF stage was an independent prognostic factor for PDAC. Our data revealed that all patients with the NBF1 stage carried an increased risk of cancer‐specific mortality than the NBF0 stage, except for those with IB stage. When taking the IA‐NBF0 stage as a reference, the HRs of cancer‐specific mortality in stage IB‐NBF0, and stage IB‐NBF1 were 3.460 and 3.766, respectively, which confirmed no significant difference. This was due to statistical bias caused by small data volume (*n* = 105, 115).

Most notably, several TNM‐NBF1 stages exhibited higher HRs than TNM‐NBF0 stages with higher TNM stages. As shown in our results, the cancer‐specific mortality of stage IB‐NBF0 showed no significant difference in comparison with stage IIB‐NBF0. In addition, the cancer‐specific mortality was remarkably higher in stage IB‐NBF1 patients than in stage IIA‐NBF0 patients. These findings illustrated the limitations of the TNM staging system in clinical practice. NBF stages should serve as a main supplementary part to the traditional AJCC‐TNM staging system and could make improvements in clinical management and prognostic prediction of PDAC.

Previous studies on colon and rectal cancer revealed that a few node‐negative TNM stages with NBF1 exhibited poorer prognosis than several node‐positive TNM stages with NBF0.^19^, ^20^ Similarly, a higher risk of cancer‐specific mortality was also discovered in stage IIB‐NBF1 than stage III‐NBF0 patients in our study. We attributed this phenomenon to the frequent clinical application of neoadjuvant chemotherapy. Stage III patients are usually locally advanced at diagnosis and not qualified for instant surgeries. Pathological biopsy and neoadjuvant chemotherapy are recommended for these patients.^41^ In addition, neoadjuvant chemotherapy could serve as a more rational choice for those with borderline resectable PDAC and even resectable PDAC. A national retrospective study in French proved that a positive margin was more likely to occur in borderline resectable PDAC patients without neoadjuvant chemotherapy.^42^ An international, multicenter, retrospective study further demonstrated that patients with PDAC of the pancreatic head who were candidates for venous reconstruction were supposed to receive routine neoadjuvant treatment.^43^ Moreover, a randomized controlled trial conducted in 2021 confirmed the rationality of neoadjuvant chemotherapy for patients with purely resectable PDAC of the head of the pancreas.^44^ In comparison with the instant surgery group, a significantly higher median disease‐free survival was observed in the neoadjuvant chemotherapy group. The role that neoadjuvant chemotherapy plays in the prognosis of PDAC has been increasingly recognized by more medical institutions.

As mentioned above, NBFs are closely related to the quality of chemotherapy. The analysis of the independent factors and their interaction in our study had confirmed the significance of the difference in chemotherapy between the NBF0 stage and the NBF1 stage (*p* for interaction = 0.006), which means that the rate of receiving chemotherapy was significantly higher in patients with the NBF0 stage. Hence, we concluded that those with higher TNM stage and NBF0 stage (better insurance status, marital status, county‐level median household income, and unemployment) were supposed to obtain more timely and accurate neoadjuvant chemotherapy in comparison with patients with lower TNM stage and NBF1 stage. In this respect, we speculated that the NBF stage might serve as a more powerful prognosticator for CSS than the TNM stage in the locally advanced and node‐positive status. The TNM‐NBF staging system is identified to be a more promising method in predicting the prognosis of patients with PDAC than the TNM staging system only.

However, there were several limitations in our study. First, the TNM‐NBF staging system did not cover a field for other BFs, such as carbohydrate antigen 199 (CA199), bilirubin level, and serum biomarkers, which might have an impact on CSS of PDAC.^45^, ^46^, ^47^ Second, the data of several subgroups (e.g., IA stage and IB stage) obtained from the SEER database were small, which could result in statistical bias. Moreover, our study mainly focused on a US population, and whether the NBF stage has an impact on the survival of PDAC in other countries still requires further exploration. Finally, the present study was based on retrospective analyses and prospective studies should be carried out to test the authenticity of the relationship between the NBF stage and survival.

## 5 CONCLUSIONS

The current study revealed that NBFs, including insurance status, marital status, county‐level median household income, and unemployment, were significant prognostic factors in PDAC. NBF stage was independently associated with the CSS of PDAC. In addition, the combination of the TNM stage and the NBF stage remarkably raised the prognostic‐prediction accuracy compared with the TNM stage alone, which strongly called for the clinical application of the TNM‐NBF staging system. Furthermore, the NBF1 stage patients are supposed to receive closer clinical attention and follow‐up because they are more likely to benefit from additional medical support. The novel TNM‐NBF staging system promises to make great improvements in the clinical management and prognosis of patients with PDAC.

## AUTHOR CONTRIBUTIONS

Study concepts: Chao Wang, Haoda Chen. Study design: Chao Wang, Haoda Chen, and Baiyong Shen. Data acquisition: Chao Wang. Quality control of data and algorithms: Chao Wang, Wei Xu. Data analysis and interpretation: Chao Wang, Haoda Chen. Statistical analysis: Chao Wang, Wei Xu. Manuscript preparation: Chao Wang, Xiaxing Deng. Manuscript editing: Chao Wang. Manuscript review: Wei Xu, Baiyong Shen.

## FUNDING INFORMATION

This research was supported by Ruijin Hospital Affiliated to Shanghai Jiao Tong University, School of Medicine (TMSK‐2020‐111). The funders had no role in the study design, data collection and analysis, decision to publish, or preparation of the manuscript.

## CONFLICT OF INTEREST

All authors declare no conflict of interest.

## ETHICS STATEMENT

All procedures performed in studies involving human participants were in accordance with the ethical standards of the institutional and/or national research committee and with the 1964 Helsinki declaration and its later amendments or comparable ethical standards. This article does not contain any studies with human participants or animals performed by any of the authors. It has been permitted to obtain the data from the SEER database (Reference Number 10778‐Nov2018).

## Data Availability

The data of our work are available and publicly accessible. The original data comes from the SEER database.
